# *Porphyromonas* spp., *Fusobacterium* spp., and *Bacteroides* spp. dominate microbiota in the course of macropod progressive periodontal disease

**DOI:** 10.1038/s41598-021-97057-1

**Published:** 2021-09-07

**Authors:** Sabine Yip, Manijeh Mohammadi Dehcheshmeh, David J. McLelland, Wayne S. J. Boardman, Sugiyono Saputra, Esmaeil Ebrahimie, Laura S. Weyrich, Philip S. Bird, Darren J. Trott

**Affiliations:** 1 School of Animal and Veterinary Sciences, The University of Adelaide, Roseworthy, SA 5371 Australia; 2 Australian Centre for Antimicrobial Resistance Ecology, School of Animal and Veterinary Sciences, The University of Adelaide, Roseworthy, SA 5371 Australia; 3 Zoos South Australia, Adelaide Zoo, Frome Road, Adelaide, SA 5000 Australia; 4 Genomics Research Platform, School of Life Sciences, Health and Engineering, La Trobe University, Melbourne, VIC 3086 Australia; 5 School of BioSciences, The University of Melbourne, Melbourne, VIC 3010 Australia; 6 Department of Anthropology and Huck Institutes of the Life Sciences, Pennsylvania State University, University Park, PA 16801 USA; 7 School of Biological Sciences, The University of Adelaide, North Terrace Campus, Adelaide, SA 5000 Australia; 8 School of Veterinary Science, The University of Queensland, Faculty of Science, Gatton, QLD 4343 Australia

**Keywords:** Classification and taxonomy, Bacterial genomics

## Abstract

Macropod progressive periodontal disease (MPPD) is a necrotizing, polymicrobial, inflammatory disease commonly diagnosed in captive macropods. MPPD is characterized by gingivitis associated with dental plaque formation, which progresses to periodontitis and then to osteomyelitis of the mandible or maxilla. However, the underlying microbial causes of this disease remain poorly understood. In this study, we collected 27 oral plaque samples and associated clinical records from 22 captive *Macropodidae* and *Potoroidae* individuals that were undergoing clinical examination at Adelaide and Monarto Zoos in South Australia (15 healthy, 7 gingivitis and 5 periodontitis-osteomyelitis samples). The V3-V4 region of the 16S ribosomal RNA gene was sequenced using an Illumina Miseq to explore links between MPPD and oral bacteria in these animals. Compositional differences were detected between the microbiota of periodontitis-osteomyelitis cases compared to healthy samples (p-value with Bonferroni correction < 0.01), as well as gingivitis cases compared to healthy samples (p-value with Bonferroni correction < 0.05) using Permutational Multivariate Analysis of Variance (PERMANOVA). An overabundance of *Porphyromonas*, *Fusobacterium*, and *Bacteroides* taxa was also identified in animals with MPPD compared to healthy individuals using linear discriminant analysis effect size (LEfSe; p =  < 0.05). An increased abundance of *Desulfomicrobium* also was detected in MPPD samples (LEfSe; p < 0.05)*,* which could potentially reflect differences in disease progression. This is the first microbiota analysis of MPPD in captive macropods, and these results support a polymicrobial pathogenesis of MPPD, suggesting that the microbial interactions underpinning MPPD may be more complex than previously documented.

## Introduction

Macropods, which include kangaroos and wallabies, are herbivorous marsupials in the superfamily Macropodoidea within the order Diprotodontia. The regular macropod dentition varies across species, and the dental formula is I 3/1, C 0–1/0, PM 1–2/1–2, M 4/4, reflecting adaptation to a range of foraging strategies. Incisors are used for prehension, and pre-molars and molars are used for mastication. Molar progression occurs normally in grazing and some intermediate grazer-browser species, where premolar and molar teeth progressively wear down and are shed at the rostral end of the quadrant when they cease to be functional^1,2^.

Macropod progressive periodontal disease (MPPD), commonly termed ‘lumpy jaw’, is a necrotising polymicrobial dental disease characterised by the proliferation of anaerobic bacteria. MPPD is one of the most common diseases identified in macropods, especially in captive environments. Appropriate treatment is often successful with early intervention, though recurrence is common. Euthanasia is often required in advanced cases^3–6^.

The pathogenesis of MPPD is not fully understood. Commonly reported risk factors for MPPD include stressors (e.g. cold, wet weather and overcrowding), molar progression, food impaction and plaque accumulation, faecal contamination of feed areas, age, and inappropriate diet^7–9^. Coarse feed may cause gingival injury that could allow bacterial invasion. Soft foods with insufficient abrasiveness may also lead to a lack of natural ‘toughening’ of the mucosa and affect toothwear^1–3,7^.

Periodontal disease (PD) is a common, inflammatory, oral disease recognised in humans and animals. PD is generally initiated by the accumulation of dental plaque—a diverse biofilm of commensal microorganisms—that adhere to teeth. In humans, primary colonizers, such as *Streptococci* spp. and *Corynebacterium* spp. adhere to tooth enamal, followed by secondary colonisers, such as *Fusobacterium* spp*.*, which provide a foundation that can allow late-colonising bacteria, including anaerobic gram-negative bacteria, to attach^10,11^. Gingivitis, a reversible form of PD, presents as inflammation of gingivae resulting from the host’s innate and adaptive immune response to bacterial toxins, including enzymes, structural components and leukotoxins. Periodontitis, an irreversible form of PD, progresses to loss of integrity of the gingival epithelium and inflammation of the periodontal ligament, absorption of alveolar bone, tooth mobility and eventual tooth loss^4–6^.

Unlike PD in humans, MPPD commonly progresses to necrotising osteomyelitis of the mandible or maxilla, with formation of sequestra and proliferation of subperiosteal bone subsequently leading to bone deformity in the jaw. Although the pathogenesis of PD in macropods is considered to be similar to that for humans^8^, the progression to osteomyelitis, suppurative inflammation and necrosis of adjacent soft tissues observed in macropods is rare in humans^3^.

Various hypotheses have been offered to describe the pathogenesis of PD, mostly in humans. The ‘non-specific plaque’ hypothesis states that PD progression correlates with the total quantity of bacterial plaque present, while the ‘specific plaque’ hypothesis proposes that disease correlates with the presence of specific bacterial species^12^. Polymicrobial synergy and dysbiosis working together has also been proposed as a model for PD development^13^. The models assumes that bacterial interactions can be antagonistic or synergistic and that a change in the local environment initiates disease that leads to an imbalance or dysbiosis within the microbial community.

A shift in the microbial community that favours survival and toxin production by some bacteria is likely to result in polymicrobial disease. A further model, the ‘keystone-pathogen hypothesis,’^13^ contends that disease depends on the presence of a single species or group of bacteria that allows environmental change and dysbiosis to occur. This hypothesis suggests that the immune response is subverted by specific keystone-bacteria to allow progression of disease^14^. *Poryphomonas gingivialis* is considered to be the keystone pathogen in human PD^12,13,15^. In animals, a closely related pathogen *Porphyromonas gulae*, and in marsupials specifically, the recently-described *Porphyromonas loveana*, may act as similar keystone pathogens^16^.

In macropods, culture-dependent methods have been used to isolate bacterial species from the oral cavity, including those involved in MPPD. It has been reported that the major causative agents of MPPD are bacteria within the *Norcardia, Actinomyces, Bacteroides,* and *Fusobacterium* genera. *Fusobacterium necrophorum* has been the most commonly isolated bacteria from MPPD lesions^8,17,18^. However, there have been cases of MPPD presumed to be attributable to *Bacteroides,* with no *Fusobacterium* species isolated, as well as cases where neither species were identified^19,20^. In the healthy oral cavity, aerobic Gram-positive organisms are predominant, although *Fusobacterium* species, routinely identified in human oral microbiota and PD, have typically not been isolated in macropods^8,21–23^. This striking difference between healthy and diseased groups in the study of Samuel, questioned whether *F. necrophorum* escaped detection, and is part of the endogenous micriobiota or introduced from an external source^18^.

Culture-independent studies using PCR-Denatured Gradient Gel Electrophoresis (PCR-DGGE) have detected *F. necrophorum* at different stages of MPPD, along with other Gram-negative anaerobic bacteria belonging to *Bacteroides* and *Porphyromonas* genera^3^. This finding was supported by quantitative PCR for a leucotoxin gene of *F. necrophorum*, but not all animals with disease were PCR-positive. *F. necrophorum* and its leukotoxin gene were also detected at low levels in some healthy animals^24^. Bacterial families found only in the healthy group were *Pasteurellaceae* and *Moraxellaceae*. Bacterial diversity was greater in healthy mouths than in periodontitis-osteomyelitis cases, but was highest in gingivitis cases. Analysis of bacterial community structure in diseased mouths showed that a small proportion of organisms were responsible for a large amount of functional interaction, supporting the ecological plaque hypothesis^3^. *P. loveana* and *P. gulae* were the predominant porphyromonads isolated from the macropod oral cavity^16^.

Next generation sequencing (NGS) methods can be applied to profile bacterial communities by sequencing the hypervariable regions of amplified 16S rRNA-encoding genes. This region of the rRNA gene is able to identify prokaryotes at the individual species level in some cases, and it has been used to study oral, gut and other microbiotas of animals, including macropods^25,26^. Sequencing methods have also defined the oral microbiota of Tasmanian devils (*Sarcophilus harrisii*)*,* identifying over 1000 unique microbial sequences, or operational taxonomic units (OTUs), which would have been impossible to determine using culture based methods^27^. The oral microbiota of macropods, in either health or disease, has not been studied using High Throughput Seuqencing (HTS) methods. This study uses HTS to explore the oral microbiota of healthy captive macropods compared to those diagnosed with MPPD. We hypothesise that specific changes in microbiota profile contribute to the induction and progression of MPPD. The aim of this study was to identify microbiota changes in two stages of disease (gingivitis and periodontitis-osteomyelitis) compared to the healthy condition. This will add to our understanding of the microbiology of MPPD in macropods and may lead to improved preventative measures or treatment of the disease.

## Results

### Clinical descriptions

After examination, 27 samples from 22 animals were classified as healthy (n = 15), gingivitis (n = 7) or periodontitis-osteomyelitis cases (n = 5). Supplementary 1 provides further clinical details on the two most severe cases of MPPD sampled.

### Microbiota profiling

**Figure 1:**
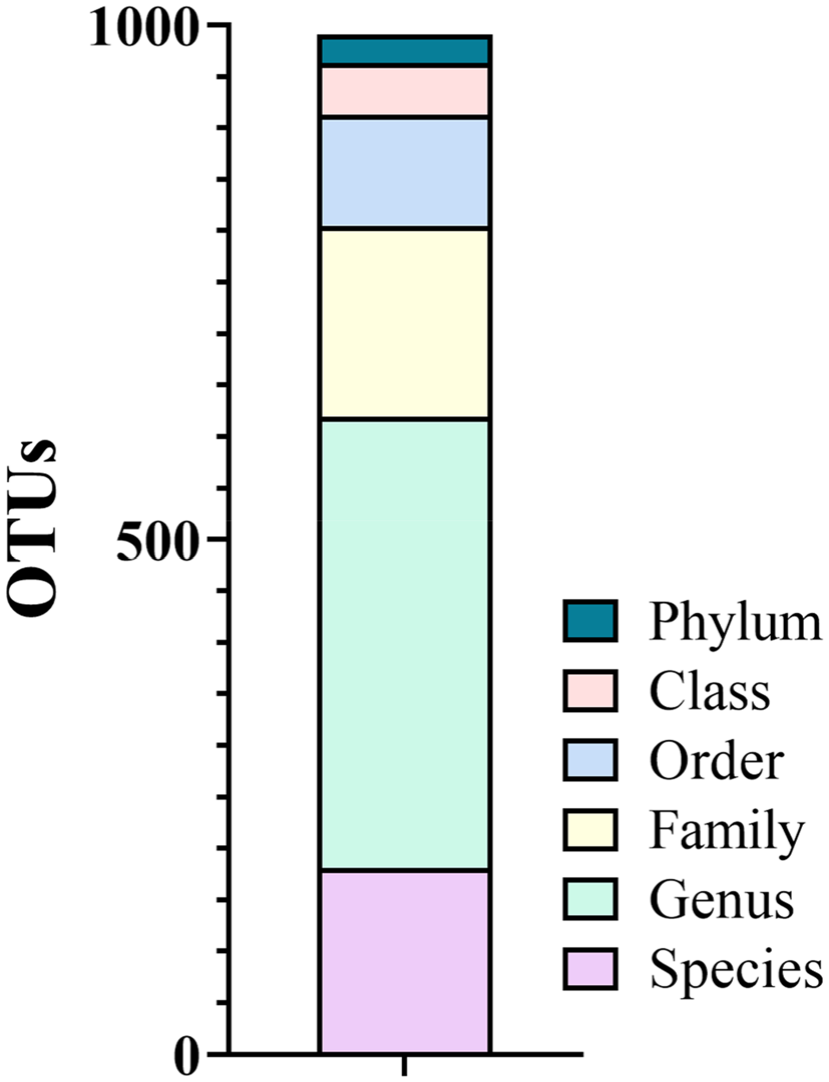
Overview of macropod oral plaque taxonomic profiling in this study. Numbers of OTUs (operational taxonomic units) taxonomically assigned to the phylum, class, order, family, genus, and species levels overall are shown.

The relative abundances of bacteria at the phylum, genus, and species taxonomic levels were explored in 27 animals belonging to healthy, gingivitis and periodontitis-osteomyelitis categories. In total, 1178 OTUs were detected, assigned to 28 phyla, 186 families, 438 genera and 181 species (Fig. 1).

**Figure 2:**
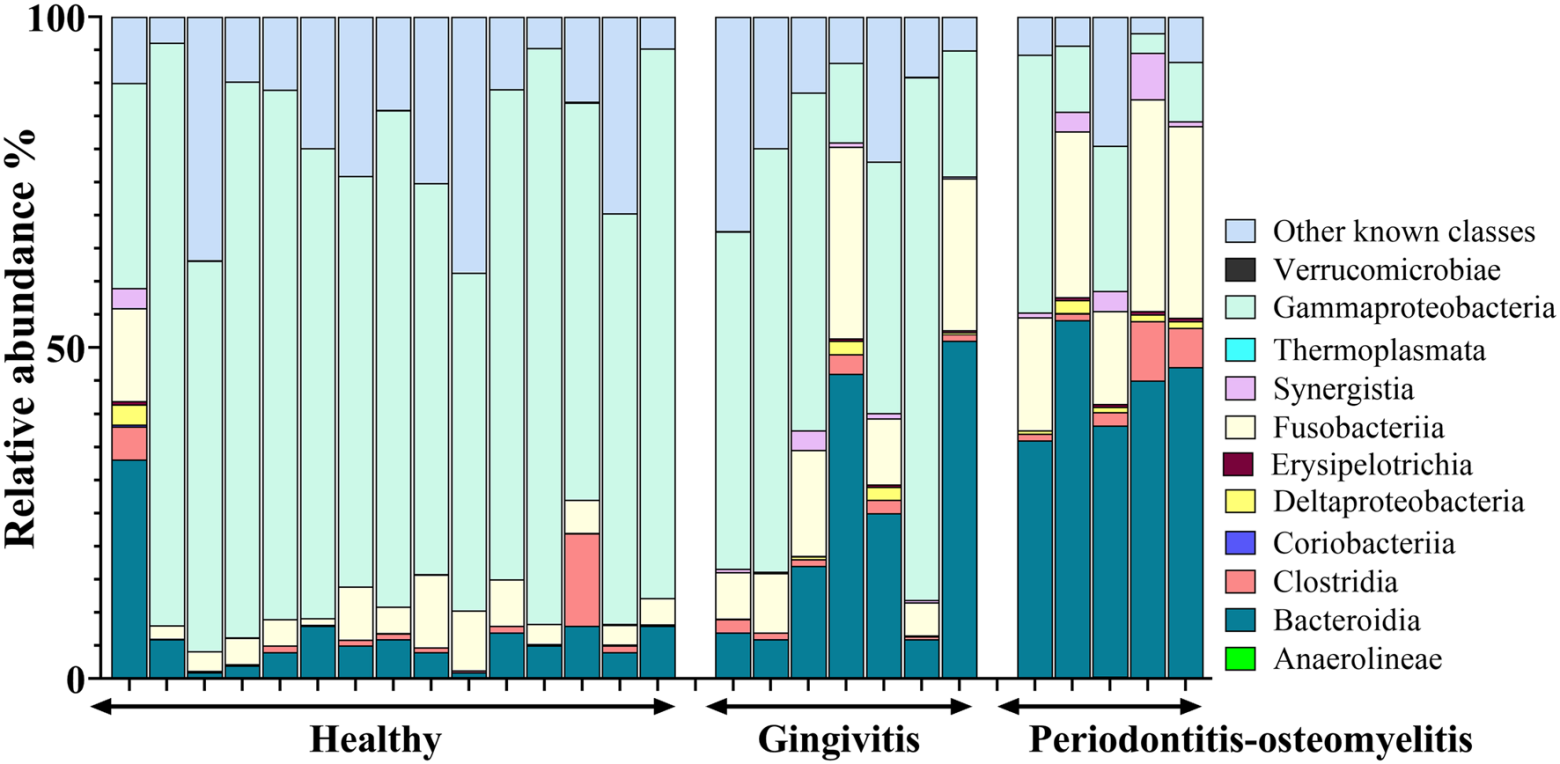
Microbiota of oral healthy macropod samples (n = 15) compared to gingivitis (n = 7) and periodontitis-osteomyelitis samples (n = 5) at the class level.

Proteobacteria and Actinobacteria were the most abundant phyla in healthy samples, forming 80.8% of the entire microbiota (Supplementary 2). We also explored microbiota identified at the class and genus levels in healthy, gingivitis, and periodontitis-osteomyelitis samples (Fig. 2 and Table 1). At the genus level, *Moraxella, Fusobacterium* and *Lautropia* were the most abundant genera across all samples (Supplementary 3).

**Table 1:** Mean and standard deviations of genera with significant changes in gingivitis vs healthy as well as periodontitis-osteomyelitis vs healthy.

	Healthy (% relative abundance)		Gingivitis (% relative abundance)		Periodontitis-osteomyelitis (% relative abundance)		Gingivitis vs healthy		Periodontitis-osteomyelitis vs healthy	
	Mean	SD	Mean	SD	Mean	SD	p-value	Over	p-value	Over
*Porphyromonas*	1.096	3.331	11.430	14.190	22.200	8.380	0.001	Gingivitis	0.001	Periodontitis-osteomyelitis
*Fusobacterium*	4.798	3.767	12.860	10.190	23.000	7.840	0.032	Gingivitis	0.001	Periodontitis-osteomyelitis
*Neisseria*	9.710	6.670	3.820	4.330	0.989	1.691	0.038	Healthy	0.008	Healthy
*Bacteroides*	0.769	2.831	4.240	5.060	14.800	9.730	0.044	Gingivitis	0.002	Periodontitis-osteomyelitis
*Fretibacterium*	0.216	0.771	0.816	0.988	1.469	1.409	0.001	Gingivitis	0.002	Periodontitis-osteomyelitis
*Lachnospiraceae_UCG-009*	0.000	0.000	0.001	0.002	0.001	0.001	0.034	Gingivitis	0.002	Periodontitis-osteomyelitis
*Tannerella*	0.218	0.771	1.002	1.368	0.691	0.312	0.001	Gingivitis	0.004	Periodontitis-osteomyelitis
*Haemophilus*	0.543	2.063	0.483	1.113	0.079	0.077	0.045	Healthy	0.009	Healthy
*Desulfomicrobium*	0.143	0.515	0.546	0.731	0.793	0.699	0.024	Gingivitis	0.002	Periodontitis-osteomyelitis
*Johnsonella*	0.099	0.205	0.436	0.306	0.383	0.148	0.003	Gingivitis	0.010	Periodontitis-osteomyelitis
*Akkermansia*	0.005	0.004	0.003	0.004	0.001	0.001	0.048	Healthy	0.023	Healthy
*Corticibacter*	0.000	0.000	0.347	0.737	0.119	0.202	0.000	Gingivitis	0.000	Periodontitis-osteomyelitis
*Ralstonia*	0.011	0.010	0.003	0.003	0.000	0.000	0.021	Healthy	0.004	Healthy
*Clostridium_sensu_stricto_1*	0.064	0.212	0.000	0.001	0.000	0.000	0.009	Healthy	0.012	Healthy
*Christensenellaceae_R-7_group*	0.050	0.128	0.080	0.090	0.295	0.334	0.015	Gingivitis	0.008	Periodontitis-osteomyelitis
*Filifactor*	0.042	0.160	0.145	0.146	0.121	0.071	0.004	Gingivitis	0.002	Periodontitis-osteomyelitis
*Rhodoferax*	0.014	0.018	0.002	0.003	0.000	0.001	0.031	Healthy	0.021	Healthy
*Atopobium*	0.023	0.089	0.013	0.022	0.019	0.030	0.019	Healthy	0.000	Healthy

LEfSe test with alpha value = 0.05 and threshold of absolute logarithmic LDA score > 2 was employed for statistical analysis.

### MPPD is associated with compositional shifts in oral microbiota, but not diversity.

**Figure 3:**
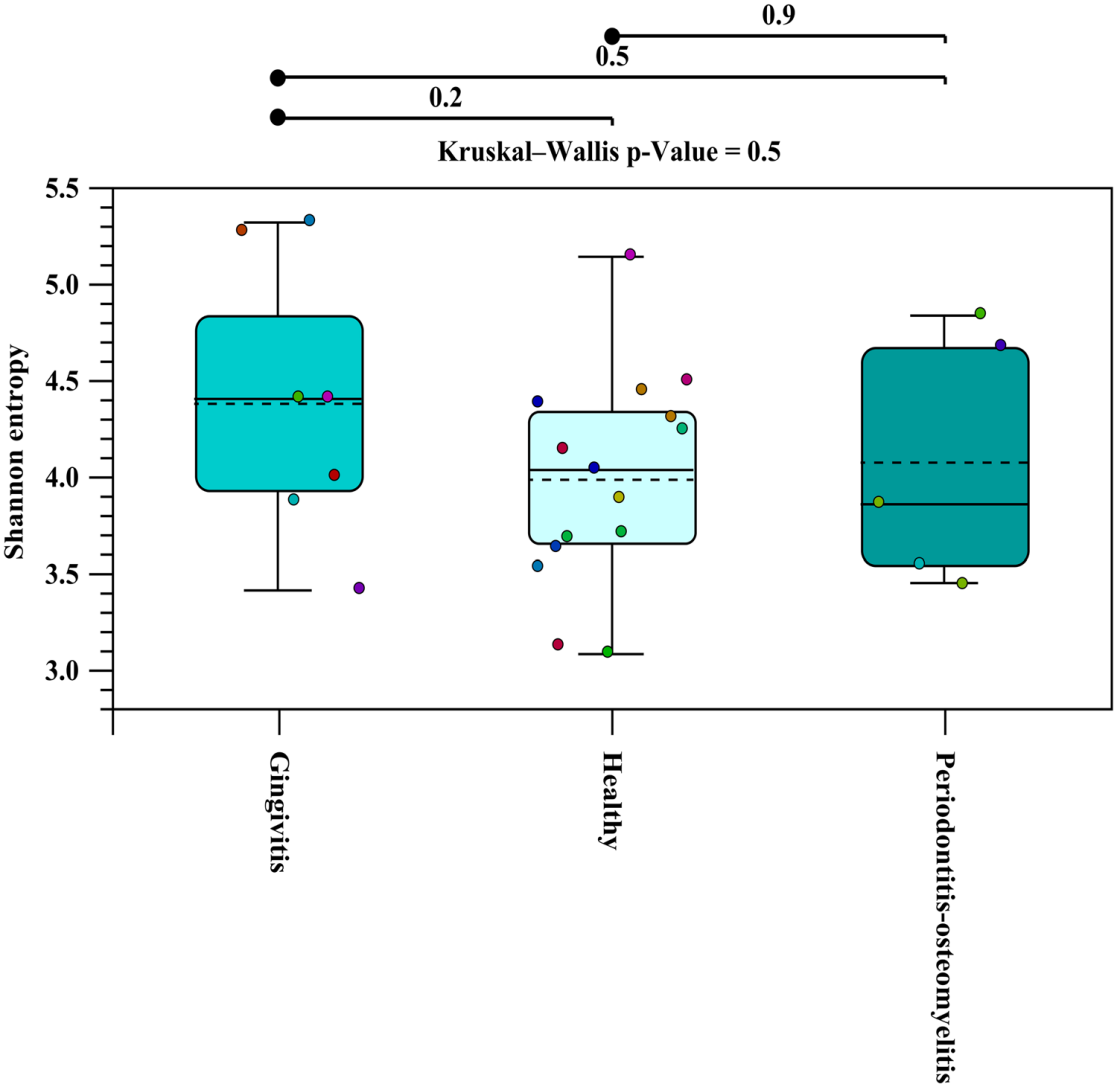
Alpha diversity was performed based on Shannon Index.

We examined if shifts in alpha diversity were apparent between health and diseased animals using the Shannon’s Diversity Index (Fig. 3). There were no significant shift in alpha diversity between healthy, gingivitis, and periodontitis-osteomyelitis sample groups (Fig. 3).

**Figure 4:**
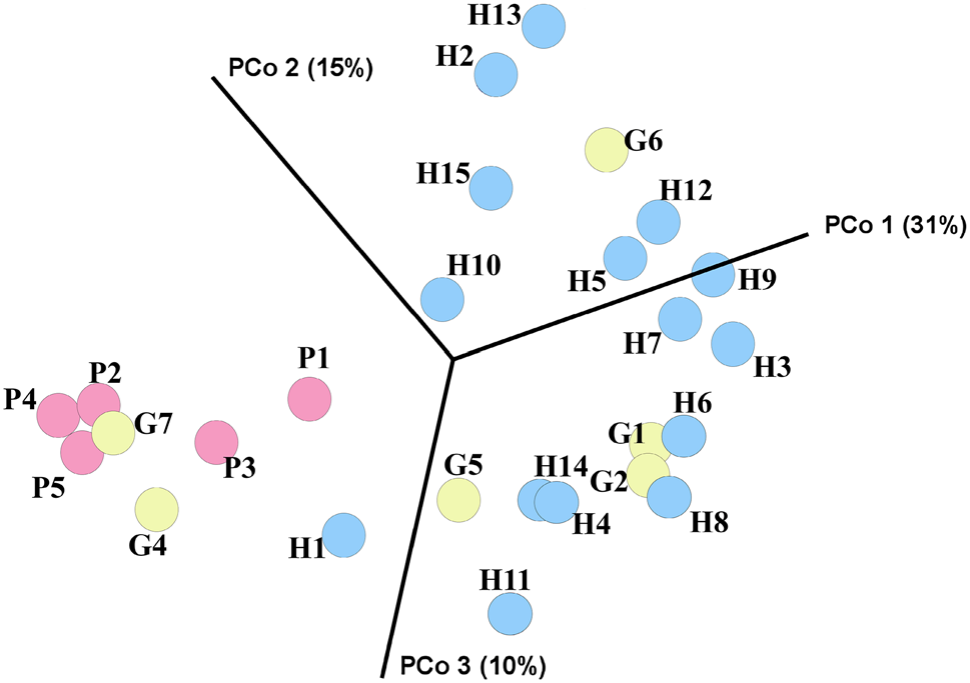
Plot of Principal Coordinate Analysis (PCoA) analysis of oral microbiota composition in periodontitis-osteomyelitis (P), gingivitis (G), and healthy (H) samples.

In contrast, the composition (beta diversity) of oral microbiota in periodontitis-osteomyelitis samples were distinguishable from the healthy and gingivitis samples using Principal Coordinate Analysis (PCoA) plot of Bray Curtis Values (Fig. 4). In a PCoA, periodontitis-osteomyelitis samples grouped to the exclusion of healthy samples on the first PCoA component of a 3D PCoA plot (Fig. 4). Supplementary 7 presents the formula PCoA components. The first component explains 31% of variance in the microbiome data (Fig. 4). The first component separated periodontitis-osteomyelitis samples from healthy and gingivitis samples, as periodontitis-osteomyelitis samples had the lowest negative coefficient values for this component (Supplementary 7). Further, the microbial composition of healthy animals was significantly different from those suffering from both periodontitis-osteomyelitis (PERMANOVA with Bonferoni correction; 0.0002) and gingivitis (p = 0.0323) (Table 2). Further, the composition of oral micorbiota in animals with periodontitis-osteomyelitis was not significantly different from those with gingivitis (p = 0.0833) (Table 2), suggesting that there may be compositional similarities between these two stages of disease. Together, these results suggest that a distinct oral microbial communities are associated with PD and gingivitis in macropods compared to healthy animals.

**Table 2:** Permutational multivariate analysis of variance (PERMANOVA) test using Bray–Curtis index showed significant differences in microbiota comparison of periodontitis-osteomyelitis group against healthy group as well as gingivitis against healthy group.

PERMANOVA analysis (Bray–Curtis)				
Group 1	Group 2	Pseudo-f statistic	p-value	p-value (Bonferroni correction)
Gingivitis	Healthy	2.2730	0.0108	0.0323
Gingivitis	Periodontitis-osteomyelitis	2.9593	0.0278	0.0833
Healthy	Periodontitis-osteomyelitis	7.4907	0.0001	0.0002

### *Porphyromonas*,* Fusobacterium* and *Bacteroides* dominate microbiota in both gingivitis and MPPD

**Figure 5:**
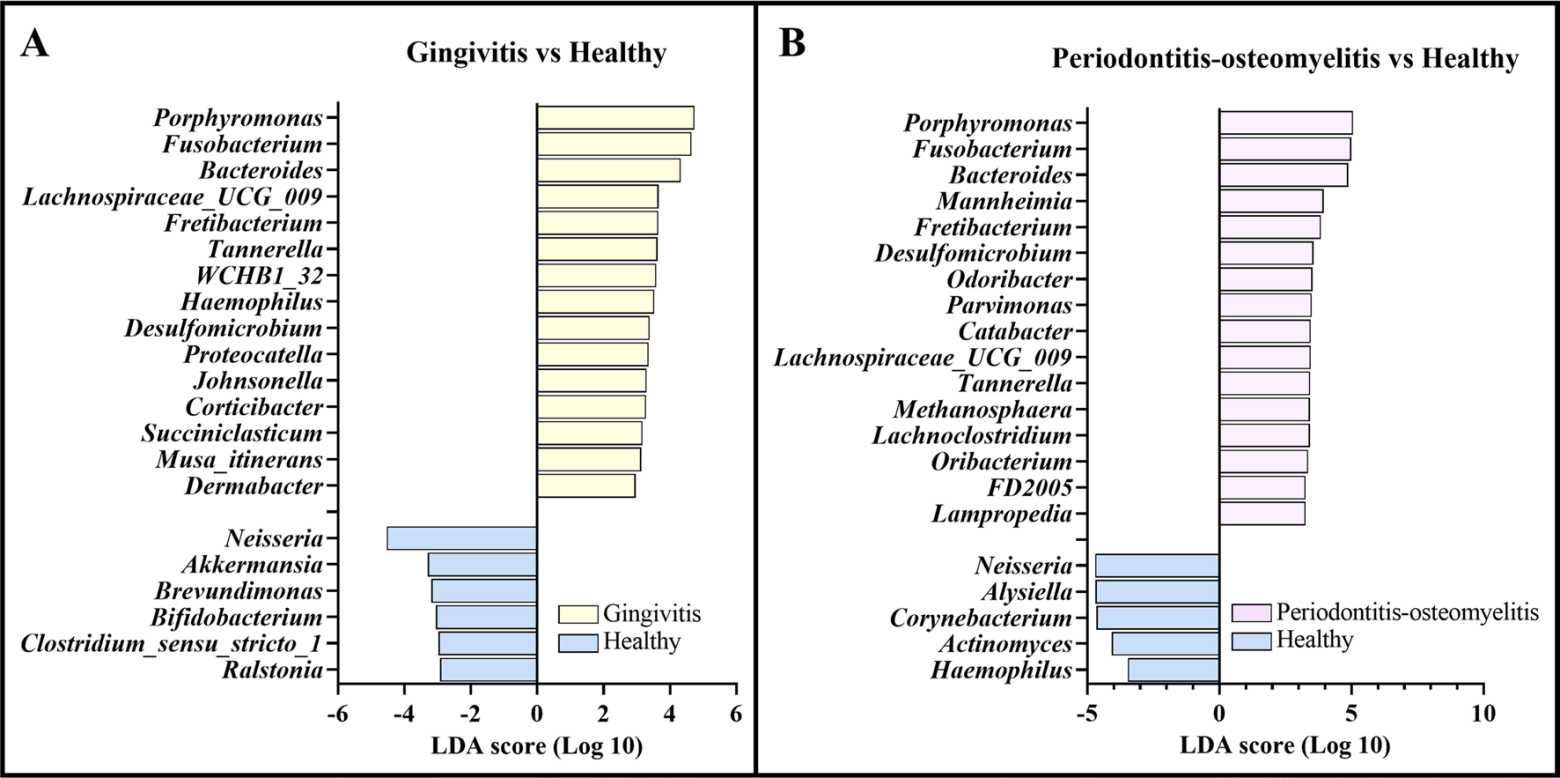
Biomarker discovery in macropod progressive periodontal disease using LEfSe test. (**A**) The main genera that differentiate gingivitis from healthy samples. (**B**) The main genera that differentiate periodontitis-osteomyelitis from healthy samples. LEfSe test with alpha value = 0.05 and threshold of absolute logarithmic linear discriminant analysis (LDA) score > 2. Higher absolute values of the LDA score represent higher enrichment and discriminative performance. Compared to healthy, *Porphyromonas*, *Fusobacterium* are overabundant in gingivitis and periodontitis-osteomyelitis samples. In contrast, *Neisseria* is underabundant in gingivitis and periodontitis-osteomyelitis samples.

A LEfSe test was utilized to identify specific taxa associated with each disease state. We observed significant differences in taxa abundance in the course of disease. The abudance of 27 genera were significantly different in healthy animals versus those suffering from gingivitis, while 66 genera were differnet between healthy and periodontitis-osteomyelitis animals (Fig. 5; Table 2; Supplementary 4 and Supplementary 5).

In the comparison of gingivitis and healthy samples, *Porphyromonas* (logarithmic LDA score = 4.743 and p-value = 0.001), *Fusobacterium* (logarithmic LDA score = 4.648 and p-value = 0.031), and *Bacteroides* (logarithmic LDA score = 4.666 and p-value = 0.044) were the dominant taxa that were overrepresented in gingivitis samples. In contrast, *Neisseria,* a genera within the Proteobacteria (the most abundant phyla in healthy samples) (logarithmic LDA score = 4.528 and p-value = 0.037) and *Akkermansia* (logarithmic LDA score = 3.297 and p-value = 0.077) were overrepresented in healthy samples (Fig. 5, Table 1, Supplementary 4).

In the comparison of periodontitis-osteomyelitis and healthy samples, *Porphyromonas* (logarithmic LDA score = 4.743 and p-value = 0.001), *Fusobacterium* (logarithmic LDA score = 4.648 and p-value = 0.031), and *Bacteroides* (logarithmic LDA score = 4.666 and p-value = 0.044) were the dominante species that were overrepresented in periodontitis-osteomyelitis, similar to the gingivitis. In contrast, *Neisseria* was again overabundant in healthy animals compared to those suffering from periodontitis-osteomyelitis (Fig. 5, Table 1, Supplementary 5).

We also investigated differences in taxa abunance in animals suffering from gingivitis or periodontitis-osteomyelitis. Eighteen genera were significantly different in abundance in both the gingivitis versus healthy and periodontitis-osteomyelitis versus healthy sample comparisons (Table 1, Supplementary 6). Of particular note, the abundance of *Desulfomicrobium* increased in both gingivitis and periodontitis-osteomyelitis in comparison to healthy samples (Table 1), with the marked increase in periodontitis-osteomyelitis samples potentially having additive effects on disease progress (logarithmic LDA score = 3.564 and p-value = 0.002) (Supplementary 5).

We confirmed these resulst by additional performing Pearson correlations on species abundance and disease. Pearson correlations revealed highly significant, positive correlations (> 72%, p < 0.01) between the relative abundance of *Porphyromonas*, *Fusobacterium*, *Bacteroides,* and *Desulfomicrobium* in oral macropod samples (Table 3). In contrast, there was negative correlation between the abundance of *Neisseria* genus with *Porphyromonas*, *Fusobacterium*, *Bacteroides*, and *Desulfomicrobium* genera (Table 3). Signature of *Porphyromonas*, *Fusobacterium*, and *Bacteroides* in MPPD was robust and consistent in different sexes, species, and zoos (Supplementary 8).

**Table 3:** Correlation between relative abanudances of *Porphyromonas*, *Fusobacterium*, *Bacteroides*, *Desulfomicrobium*, and *Neisseria* in 27 oral macropod samples.

	*Fusobacterium*	*Bacteroides*	*Desulfomicrobium*	*Neisseria*
*Porphyromonas*	Correlation = 0.851	Correlation = 0.728	Correlation = 0.721	Correlation = − 0.505
	p-value = 0.00	p-value = 0.00	p-value = 0.00	p-value = 0.007
*Fusobacterium*		Correlation = 0.860	Correlation = 0.623	Correlation = − 0.592
		p-value = 0.00	p-value = 0.001	p-value = 0.001
*Bacteroides*			Correlation = 0.443	Correlation = − 0.470
			p-value = 0.021	p-value = 0.013
*Neisseria*				Correlation = − 0.453
				p-value = 0.018

Pearson correlation coefficient and its p-value are presented.

## Discussion

Periodontal diseases are commonly reported clinical disorders in animals. Here, for the first time, the oral microbiota of healthy and MPPD-affected captive macropods were explored using 16S rRNA gene seuqencing. This study characterised the healthy macropod oral microbiota and identified differences in bacterial composition and taxa abundances between healthly samples and the different stages of MPPD, thus providing novel information about this polymicrobial disease and its prospective pathogenesis.

There are similarities and differences between PD in human and MPPD in macropods. In humans, PD is initiated by dental plaque and exhibits progression from reversible gingivitis to irreversible periodontitis. However, unlike PD in humans, MPPD commonly progresses to necrotising osteomyelitis of the mandible or maxilla, with the formation of sequestra and proliferation of subperiosteal bone subsequently leading to bone deformity in the jaw (the characteristic ‘lumpy jaw’). The progression to osteomyelitis, suppurative inflammation and necrosis of adjacent soft tissues observed in macropods is rare in humans^3,28^. In humans, early colonisers, such as *Streptococcus* spp. and *Fusobacterium* spp. provide a foundation that can allow late colonising bacteria to attach, which includes many anaerobic Gram-negative bacteria^4–6,29^. In the present study, we found that *Porphyromonas*, *Fusobacterium*, and *Bacteroides* are the most abundant genera in gingivitis samples. These genera also dominated the microbiome in periodontitis-osteomyelitis cases (the advanced stages of disease). Also, we observed a significant and positive correlation between the relative abundances of *Porphyromonas* and *Fusobacterium* genera and disease, similar to human PD. In contrast to humans, *Streptococcus* did not appear to be a major component of plaque microbiota in macropods.

It has been well-documented that *Fusobacterium nucleatum* can enhance the attachment and invasion of *Porphyromonas gingivalis* or *Aggregatibacter actinomycetemcomitans* to human gingival epithelial cells^29^. In another study, *Porphyromonas gingivalis* entry into gingival epithelial cells was modulated by *Fusobacterium nucleatum*^30^. Additionally, it has been proposed that *Fusobacterium* spp. can inhibit the initial host innate immune response^29^. Scanning electron microscopy has shown that *P*. *gingivalis* and *F. nucleatum* can form consortia and penetrate Ca9-22 cells within 30–60 min after infection (early colonisation)^30^. Altogether, we suggest that *Fusobacterium* may be involved in early colonisation in MPPD, enhancing adhesion and invasion of species within the *Porphyromonas* genus that are likely to be either *P. gulae* or *loveana.* This interplay has been observed in murine alveolar bone loss and arthritis onset^31^.

Microbiota profiling using 16S rRNA gene pyrosequencing identified the Gram-negative bacterial genera *Fusobacterium, Bacteroides,* and *Porphyromonas* as the dominant taxa in MPPD, as noted in previous culture and DGGE-based studies^3,8,17,18,20^. These findings support the hypothesis that *Porphyromonas* and *Bacteroides* may have a bigger role in disease pathogenesis than has historically been proposed. The importance of pathogens other than *F. necrophorum* in MPPD was first noted in 1977, and recent DGGE-based molecular studies, confirmed that *Porphyromonas* and *Bacteroides* are important genera in MPPD pathogenesis^3,20,24^. However, caution should be applied, as bacterial abundance may not necessarily correlate with pathogenicity.

Abundance of *Desulfomicrobium* also increased in MPPD samples (LEfSe test, p < 0.05)*,* which could potentially have additive effects on disease progress. *Desulfomicrobium orale* has also been isolated from subgingival plaque of human patients with PD^32^, identified as a human oral pathogen^33^. This is the first time that another significant genera associated with PD in humans has been reported in MPPD, albeit at low abundance when compared to *Porphyromonas, Bacteroides* and *Fusobacterium*.

The dominance of the Proteobacteria phylum found in the oral microbiota of healthy macropods was similar to another study of the salivary microbiota in Tammar wallabies^25^. This contrasts with previous reports on the oral microbiota of other marsupials, such as Tasmanian Devils, which have a similar proportion of Proteobacteria, Bacteroidetes, Fusobacteria, and Firmicutes phyla (at around 20% of each) and koalas, where Bacteroidetes and Firmicutes were in the top three phyla^27,34^. In macropods, the highly abundant Proteobacteria phylum is composed of many species associated with gingival health in comparison to either gingivitis or periodontitis-osteomyelitis^3^. In particular, Pasteurellaceae and Moraxellaceae were the two major families found to be abundant in healthly samples^3^. An increased abundance of Gram-positive non-sporulating rods such as *Corynebacterium *sp. and *Actinomyces *sp. in healthy compared to diseased samples has also been reported in culture-based studies^18,23^. In line with those studies, species in the genera *Corynebactrium, Actinomyces, Streptococcus, Lautropia, Leptotrichia* and *Capnocytophaga* are associated with the healthy oral cavity in human microbiota studies^35^. It has also been noted that some disease-associated genera have overlapping species that are also present in healthy samples^35^.

Despite being the first study to profile the microbiota of MPPD using 16S rRNA gene pyrosequencing, this study had some limitations. In microbiota studies, sequencing depth normally includes the family, genus and sometimes species level, but there can be many OTUs that cannot be identified at the lowest level. The current study was based on a single zoological collection of animals at two sites, and the availability of MPPD cases was a limiting factor on sample size. Subsequently, there was an unequal number of healthy, gingivitis and periodontitis-osteomyelitis cases available for study, with only a limited number of gingivitis and periodontitis-osteomyelitis samples. Additionally, the low sample size meant that different species of macropods were included together in the study and the yellow-footed rock wallaby (YFRW) was overrepresented. Larger sample sizes, and comparison between macropod species and zoological collections, would be beneficial in future studies.

## Conclusion

Despite individual-to-individual variation, bacterial communities likely undergo largely conserved changes during PD^36^. For the first time, we have profiled the shift in oral microbiota of captive macropods at different stages of MPPD, as well as characterised the healthy gingival microbiome. *Porphyromonas*, *Fusobacterium*, *Bacteroides*, and *Desulfomicrobium* may play key roles in this disease, as they appear at higher prevalence in MPPD cases compared with healthy animals. Overall, these results support a polymicrobial pathogenesis of MPPD and suggest that the diversity of bacteria involved, and the interactions between them, may be more complex than has been documented previously. Improving our understanding of the pathogenesis of MPPD is key to the development of more effective preventative and therapeutic measures.

## Materials and methods

### Ethics

All experiments and experimental protocols were approved by Zoos South Australia Animal Welfare and Ethics Committee, Australia. All experiments were performed in accordance with relevant guidelines and regulations of Zoos South Australia as well as School of Animal and Veterinary Sciences, The University of Adelaide. The study was carried out in compliance with the ARRIVE guidelines (https://arriveguidelines.org).

### Selected species, clinical assessment and sample collection

Periodontal plaque samples were collected opportunistically by veterinarians during routine preventative medicine health checks or investigation of clinical oral disease from 22 captive individuals of the *Macropodidae* and *Potoroidae* families at Adelaide Zoo (AZ; n = 7) and Monarto Zoo (MZ; n = 15) in South Australia. Species sampled included Yellow-footed rock wallaby (YFRW, *Petrogale xanthopus* n = 19), Black-flanked rock wallaby *(*BFRW, *P. lateralis,* n = 1), Long-nosed potoroo (LNP, *Potorous tridactylus*, n = 1) and Tammar wallaby (TW, *Notamacropus eugenii,* n = 6). Most were captive born at AZ or elsewhere in South Australia and had been resident at their respective zoo from < 1 to 11 years. At AZ, wallabies were maintained on a grassed enclosure, supplemented with kangaroo pellets (Wombaroo Food Products, Glen Osmond, South Australia), lucerne chaff, browse, carrots and spinach. The LNP was fed a mixed diet of dog kibble, kangaroo pellets, vegetables, fruits and seeds. At Monarto, an open range zoo, wallabies were maintained in large enclosures with native vegetation, and fed ad lib meadow hay, with supplemental kangaroo pellets, lucerne chaff, and browse. Meta-data of experiment is presented at Supplementary 9.

Animals were anaesthetised with gaseous isoflurane in oxygen via face mask. Two subgingival plaque samples were collected from different quadrants of the mouth using sterile cuvettes, or whole teeth collected in the case of extraction. Animals with clinical disease were only included if they were sampled prior to commencement of antimicrobial treatment. Samples were placed immediately in transport media (10% glycerol in Wilkins Chalgren broth) and stored at either − 80 °C (AZ) or − 20 °C (MZ). Samples were transported on dry ice to the University of Adelaide, School of Animal and Veterinary Sciences, and stored at − 80 °C until analysis.

Medical records (including dental charts and diagnostic imaging where available) were used to classify oral health status at the time of sampling. Animals were classified similarly to Antiabong et al*.*^24^ as healthy, gingivitis and periodontitis-osteomyelitis cases, based predominantly on dental examination, with gingivitis and periodontitis-osteomyelitis cases respresenting early and advanced cases of MPPD, respectively. Individuals with gingivitis had gross swelling, redness of the gums, bleeding on swabbing, minor gingival recession and/or early periodontal pocket formation. Periodontitis-osteomyelitis was defined here as periodontitis with or without more progressive disease associated with soft tissue and/or bone involvement. Individuals were classified with periodontitis-osteomyelitis if, in addition to gingivitis, they had severe gingival recession, deep periodontal pockets, tooth mobility, bone necrosis, and/or other more severe lesions. Healthy animals were classified by the absence of the above gross lesions. For MPPD cases, the sample analysed was that collected from the affected tooth. Additional findings such as plaque in healthy animals (in the absence of gingivitis), other disease conditions present, episodes of previous dental disease and pouch status were also recorded.

### DNA extraction

The periodontal plaque samples were thawed at 37 °C in an anaerobic chamber (Coy, Grasslakes, Michigan, USA) and then vortexed for 20 s. DNA extraction was performed using the QIAGEN DNeasy Blood and Tissue Kit (Qiagen Hilden, Germany) spin-column protocol, modified to improve DNA yield by using 400 µL of sample, 40 µL of Proteinase K, 400 µL buffer AL and 400 µL ethanol. The elution step was performed twice, using 70µL and then 30 µL of elution buffer. Sample DNA concentrations and quality were tested using Nanodrop 1000 spectrophotometry (Thermo Fisher, Waltham, Massachusetts, USA). Concentrations were also measured with a Qubit Fluorimeter, following the assay preparation instructions from Qubit dsDNA HS Assay Kits (Thermo Fisher).

### Illumina Miseq 16S rRNA gene sequencing

Published primers were used for the amplification of the 550 bp V3-V4 region of the bacterial 16S rRNA gene. PCR products were visualised following electrophoresis in agarose and staining with Gel Red™ to confirm positive yield for each sample. Samples were submitted to the South Australian Health & Medical Research Institute DNA Sequencing Facility for 16S Microbiota library preparation and sequencing. Library preparation followed the Illumina library preparation protocol, with the following primers: forward CCTACGGGNGGCWGCAG, reverse GACTACHVGGGTATCTAATCC. Sequencing was carried out by Illumina Miseq V3 SBS Chemistry targeting machine. Amplicons were sequenced as paired reads with the length of 300 bp (2 × 300 bp).

### Microbiota profiling

Adapter trimming, fixed length trimming, merging paired reads and filtering based on the number of reads (to remove the samples with low coverage) were performed to obtain high quality sequence reads with enough depth for microbiota profiling and comparison as previously described^37^. CLC Microbial Genomics Module (QIAGEN) Version 11 was used to assign taxonomy to the reads from different samples. To this end, reads were clustered using representative sequences of pseudo-species called OTUs (operational taxonomic units). The OTU clustering tool clusters the reads and reduces the read collection in each sample to representative sequences (cluster centroids) that are 97% similar to any member of the cluster they represent. The SILVA database was used as the reference of 16S rRNAs^38^. The number of reads assigned to each OTU and the relative abundance of each OTU was calculated^39^.

### Statistical analysis

Linear discriminant analysis (LDA) effect size (LEfSe)^40^ algorithm, a test for high-dimensional biomarker discovery in metagenomic data, was used to find differentially abundant bacteria between periodontitis-osteomyelitis versus healthy as well as gingivitis versus healthy samples. The following criteria were used for selection of bacteria with statistically different relative abundance: alpha value = 0.05 and threshold of absolute logarithmic LDA score > 2. Analysis was performed in the Galaxy platform (https://huttenhower.sph.harvard.edu/galaxy/).

LEfSe algorithm benefits from a range of tests: (1) Non-parametric factorial Kruskal–Wallis (KW) sum-rank test to find bacteria with statistical significant differences between groups; (2) a set of pairwise tests among subclasses using the (unpaired) Wilcoxon rank-sum test; and (3) LDA for estimating the effect size of each differentially abundant bacteria as well as feature selection (dimension reduction)^40^.

Alpha diversity estimates the diversity within samples. Alpha diversity was performed based on Shannon Index. Kruskal–Wallis H test was used for measuring the statistical significance of alpha diversity. Kruskal–Wallis H assesses whether the values originate from the same distribution or whether their distribution is different depending on the group they belong to. This test is a nonparametric alternative to ANOVA. A significant p-value for the Kruskal–Wallis test means that at least one group has a different distribution. However, Kruskal–Wallis does not report which pairs have different distributions. Mann–Whitney U test was used to performs a pair-wise test to specifically find which pairs of groups follow different distributions.

Beta diversity measures the change in diversity between groups. Beta diversity was calculated in using CLC Microbial Genomics Module in two steps: (1) estimating the distance between each pair of samples; and (2) performing Principal Coordinate Analysis (PCoA) on the distance matrices. Bray–Curtis measurement was used to calculate the distance matrices.

PERMANOVA test^41^, also known as non-parameteric MANOVA, measures the effect of size and significance on beta diversity in comparisons of gingivitis group versus healthy and periodontitis-osteomyelitis versus healthy samples. PERMANOVA obtains its significance from a permutation test. The number of permutations was set to 99,999. Analysis was performed by CLC Microbial Genomics Module.

## Supplementary Information

Supplementary Information.
