# Supplementary material for: Porphyromonas spp., Fusobacterium spp., and Bacteroides spp. dominate microbiota in the course of macropod progressive periodontal disease
Source: Sci Rep. 2021 Sep 7;11:17775. doi: 10.1038/s41598-021-97057-1 (PMC8423738; doi:10.1038/s41598-021-97057-1)
Supplement: Supplementary file 1 — Supplementary Information. [file 41598_2021_97057_MOESM1_ESM.pdf]

Supplementary 1-9

Porphyromonas spp., *Fusobacterium* spp., and *Bacteroides* spp. dominate microbiota in the course of macropod progressive periodontal disease

Sabine Yip[1*], Manijeh Mohammadi Dehcheshmeh[2*], David J McLelland[1,3], Wayne S J Boardman[1], Sugiyono Saputra[1], Esmaeil Ebrahimie[2,4,5ɸ], Laura S. Weyrich[6,7], Philip S Bird[8], Darren J Trott[2ɸ]

[1] School of Animal and Veterinary Sciences, The University of Adelaide, Roseworthy, South Australia 5371, Australia
[2] Australian Centre for Antimicrobial Resistance Ecology, School of Animal and Veterinary Sciences, The University of Adelaide, Roseworthy, South Australia 5371, Australia
[3] Zoos South Australia, Adelaide Zoo, Frome Road, Adelaide, South Australia 5000, Australia
[4] Genomics Research Platform, School of Life Sciences, Health and Engineering, Melbourne, Victoria, 3086, Australia
[5] School of BioSciences, The University of Melbourne, Victoria 3010, Australia
[6] Department of Anthropology, Pennsylvania State University, University Park, PA, USA 16801
[7] School of Biological Sciences, The University of Adelaide, North Terrace Campus, Adelaide, South Australia, 5000, Australia
[8] School of Veterinary Science, The University of Queensland, Faculty of Science, Gatton, QLD 4343, Australia

[*]These authors contributed equally
[ɸ] Correspondence to: E.Ebrahimie@latrobe.edu.au; darren.trott@adelaide.edu.au

Supplementary 1.

(A-C) Post-mortem CT scans of a 9.5 years old female *Petrogale xanthopus* (yellow-footed rock wallaby) with severe gingivitis which progressed to periodontitis-osteomyelitis. It first presented with gingival recession and enlarged periodontal pockets of the first molar in all four quadrants, which exhibited marked buccal drift without tooth mobility. Two months later she represented with acute facial swelling in the right cheek, loose stools, and severe weight loss. Teeth in the upper right quadrant were extracted, along with a large bony sequestrum. Necrotic bone was present in the right mandible, which was fractured. (A) the right dorso-lateral skull. (B) the right skull and molars. (C) the left skull and molars. Missing teeth and bone can be seen on the right mandible. Alveolar bone resorption is seen on multiple teeth in all quadrants, exposing the tooth roots.

(D-F) Radiographs of a 4.25 years old female *Petrogale xanthopus* (yellow-footed rock wallaby) with periodontitis-osteomyelitis. It had molar extractions due to a dental abscess under the right eye two years prior. When sampled, it presented with excess plaque/tartar build up on the left quadrants, with gingival recession on the left upper PM3 and M1, which were extracted. It was classified as having periodontitis-osteomyelitis in the upper left quadrant, and gingivitis in the lower left. Four months after sampling, with repeated treatment, the extraction site had healed, however, two more M1 teeth were extracted. (D) Oblique view of skull isolating the left mandible, and (E) intra-oral dental radiograph of the left mandible pre-extraction. (F) After removal of retained root.

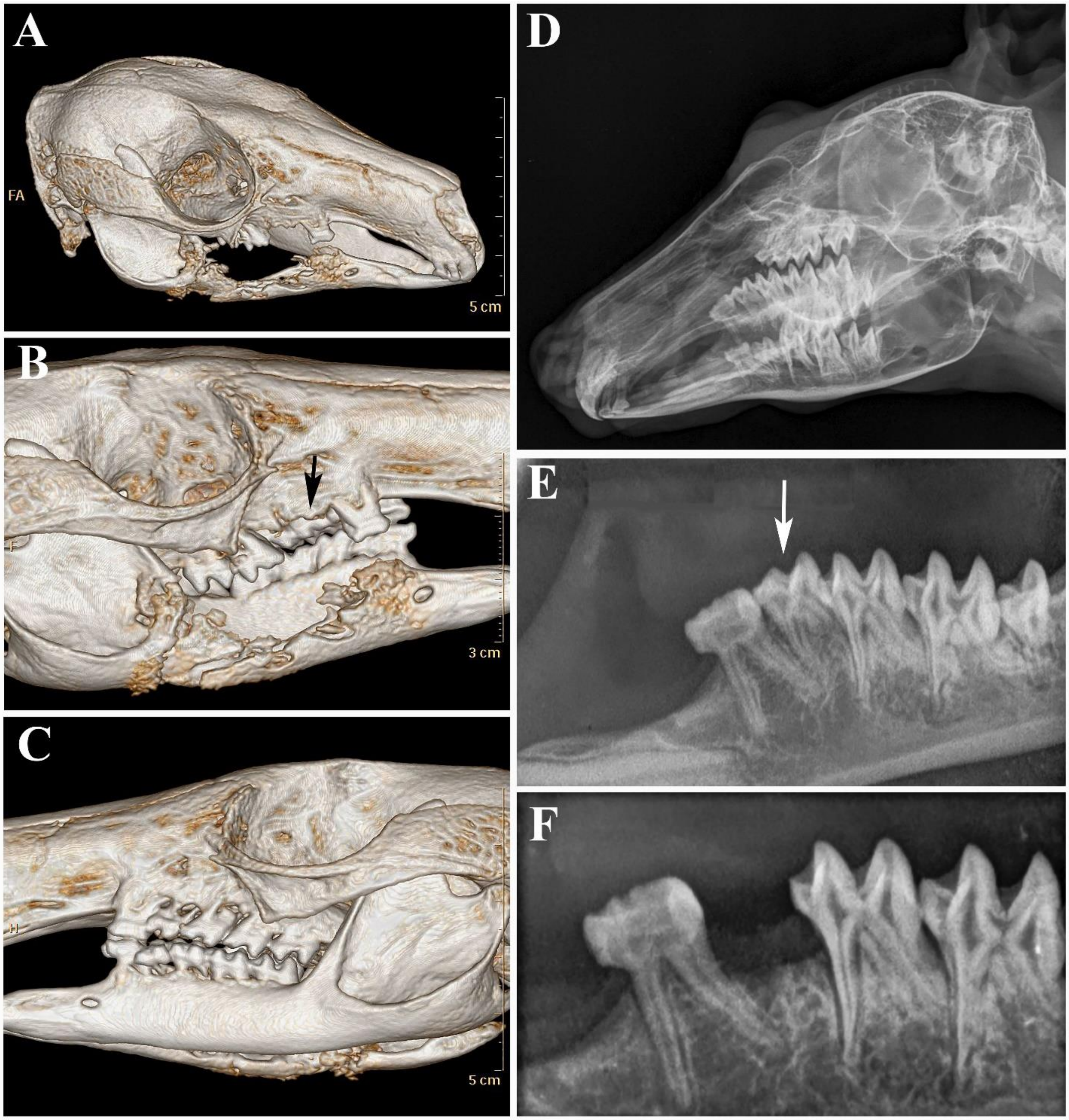
A
FA
5 cm
B
3 cm
C
5 cm
D
E
F

Supplementary 2. Microbiota of healthy macropod in phylum level.

Phylum	Mean	StDev	Median
Proteobacteria	0.686	0.1516	0.71
Actinobacteria	0.122	0.1039	0.1
Bacteroidetes	0.068	0.0762	0.05
Fusobacteria	0.05467	0.03623	0.04
Firmicutes	0.02813	0.03861	0.01
Patescibacteria	0.01843	0.01674	0.00853
Epsilonbacteraeota	0.01568	0.01911	0.00731
Synergistetes	0.0022	0.0077	0.00005
Spirochaetes	0.000972	0.002742	0
Cyanobacteria	0.000528	0.00085	0.000166
Verrucomicrobia	0.000169	0.000295	0.000094
Fibrobacteres	0.000155	0.000599	0
Chloroflexi	0.00008	0.000242	0
Planctomycetes	0.000039	0.000103	0
Tenericutes	0.000031	0.000064	0
Ambiguous_taxa	0.00003	0.000097	0
Acidobacteria	0.00003	0.000057	0
Euryarchaeota	0.000028	0.000109	0
Deinococcus-Thermus	0.000011	0.000035	0
Dependentiae	0.000011	0.00003	0
Gemmatimonadetes	0.000009	0.000023	0
Armatimonadetes	0.000005	0.000021	0
Omnitrophicaeota	0.000005	0.000019	0
Elusimicrobia	0.000004	0.000017	0
FBP	0.000004	0.000014	0
Rokubacteria	0.000004	0.000015	0
Thermotogae	0.000003	0.00001	0
Lentisphaerae	0.000002	0.000009	0

Supplementary 3. Relative abundance of the 30 most quantitatively dominant bacterial genera in oral plaque samples of captive macropods.

	**Overall**			**Healthy**			**Gingivitis**			**Periodontitis-osteomyelitis**		
**Genera**	Mean	StDev	Median	Mean	StDev	Median	Mean	StDev	Median	Mean	StDev	Median
Moraxella	0.134	0.111	0.110	0.132	0.095	0.140	0.196	0.148	0.210	0.054	0.040	0.040
Fusobacterium	0.103	0.096	0.050	0.048	0.038	0.030	0.129	0.102	0.100	0.230	0.078	0.250
Lautropia	0.097	0.153	0.005	0.154	0.183	0.080	0.037	0.074	0.001	0.011	0.012	0.009
Porphyromonas	0.077	0.115	0.010	0.011	0.033	0.001	0.114	0.142	0.020	0.222	0.084	0.190
Neisseria	0.066	0.065	0.040	0.097	0.067	0.100	0.038	0.043	0.020	0.010	0.017	0.004
Corynebacterium	0.055	0.083	0.030	0.084	0.103	0.050	0.025	0.019	0.020	0.012	0.016	0.006
Alysiella	0.049	0.095	0.008	0.081	0.119	0.010	0.014	0.010	0.010	0.001	0.001	0.001
Bacteroides	0.043	0.073	0.001	0.008	0.028	0.000	0.042	0.051	0.030	0.148	0.097	0.130
Bergeyella	0.035	0.028	0.020	0.031	0.021	0.020	0.042	0.035	0.030	0.040	0.042	0.020
Brachymonas	0.033	0.040	0.010	0.049	0.046	0.040	0.020	0.022	0.007	0.005	0.003	0.005
Campylobacter	0.019	0.022	0.010	0.016	0.019	0.007	0.030	0.032	0.010	0.013	0.009	0.010
Actinomyces	0.023	0.027	0.010	0.029	0.029	0.020	0.019	0.029	0.010	0.010	0.017	0.003
Conchiformibius	0.012	0.021	0.003	0.013	0.024	0.003	0.016	0.022	0.007	0.003	0.003	0.001
Rothia	0.009	0.034	0.000	0.000	0.001	0.000	0.035	0.063	0.001	0.000	0.000	0.000
Capnocytophaga	0.008	0.011	0.004	0.006	0.008	0.003	0.006	0.003	0.007	0.013	0.021	0.003
Mannheimia	0.007	0.014	0.000	0.003	0.010	0.000	0.005	0.008	0.000	0.020	0.021	0.020
Leptotrichia	0.007	0.011	0.001	0.007	0.011	0.003	0.010	0.014	0.001	0.001	0.001	0.001
Streptococcus	0.007	0.009	0.005	0.009	0.011	0.006	0.005	0.002	0.005	0.002	0.001	0.002
Fretibacterium	0.006	0.010	0.001	0.002	0.008	0.000	0.008	0.010	0.005	0.015	0.014	0.007
Lampropedia	0.005	0.017	0.000	0.000	0.000	0.000	0.017	0.031	0.001	0.003	0.004	0.001
Pasteurella	0.005	0.008	0.003	0.007	0.011	0.004	0.002	0.002	0.001	0.004	0.005	0.001
Tannerella	0.005	0.009	0.000	0.002	0.008	0.000	0.010	0.014	0.005	0.007	0.003	0.008
Haemophilus	0.004	0.016	0.000	0.005	0.021	0.000	0.005	0.011	0.000	0.001	0.001	0.001
Tessaracoccus	0.004	0.006	0.001	0.005	0.008	0.003	0.003	0.003	0.001	0.001	0.002	0.001
Luteimonas	0.004	0.009	0.000	0.004	0.010	0.000	0.001	0.002	0.000	0.008	0.012	0.005

Proteocatella	0.004	0.005	0.003	0.002	0.003	0.000	0.007	0.006	0.006	0.005	0.002	0.005
Desulfomicrobium	0.004	0.006	0.000	0.001	0.005	0.000	0.005	0.007	0.003	0.008	0.007	0.006
Treponema 2	0.003	0.007	0.000	0.001	0.003	0.000	0.004	0.011	0.000	0.006	0.009	0.000
Acinetobacter	0.003	0.004	0.001	0.003	0.005	0.001	0.004	0.004	0.004	0.001	0.002	0.000
Pyramidobacter	0.003	0.013	0.000	0.000	0.000	0.000	0.000	0.000	0.000	0.015	0.031	0.000

Supplementary 4. Genera with significant changes in gingivitis (G) versus healthy (H), according to LEfSe test with alpha value=0.05 and threshold of absolute logarithmic linear discriminant analysis (LDA) score>2.

Number	Genus	Overrepresented in Gingivitis (G) or Healthy (H)	LDA score	p-value
1	*Porphyromonas*	G	4.743	0.001
2	*Fusobacterium*	G	4.648	0.032
3	*Neisseria*	H	4.528	0.038
4	*Bacteroides*	G	4.329	0.044
5	*Lachnospiraceae_UCG_009*	G	3.658	0.034
6	*Fretibacterium*	G	3.648	0.001
7	*Tannerella*	G	3.627	0.001
8	*WCHB1_32*	G	3.587	0.034
9	*Haemophilus*	G	3.533	0.045
10	*Desulfomicrobium*	G	3.387	0.024
11	*Proteocatella*	G	3.366	0.036
12	*Akkermansia*	H	3.297	0.048
13	*Johnsonella*	G	3.293	0.003
14	*Corticibacter*	G	3.286	0.000
15	*Brevundimonas*	H	3.187	0.037
16	*Succiniclasticum*	G	3.177	0.034
17	*Musa_itinerans*	G	3.137	0.034
18	*Bifidobacterium*	H	3.044	0.037
19	*Dermabacter*	G	2.968	0.034
20	*Clostridium_sensu_stricto_1*	H	2.967	0.009
21	*Ralstonia*	H	2.926	0.021
22	*Mogibacterium*	G	2.859	0.032
23	*Filifactor*	G	2.851	0.004
24	*Suttonella*	G	2.787	0.041
25	*Rhodoferax*	H	2.776	0.031
26	*Christensenellaceae_R_7_group*	G	2.551	0.015
27	*Atopobium*	H	2.484	0.019

Supplementary 5. Genera with significant changes in periodontitis-osteomyelitis (P) versus healthy (H), according to LEfSe test with alpha value=0.05 and threshold of absolute logarithmic linear discriminant analysis (LDA) score>2.

Number	Genus	Overrepresented in periodontitis-osteomyelitis (P) or Healthy (H)	LDA score	p-value
1	*Porphyromonas*	P	5.062	0.001
2	*Fusobacterium*	P	4.987	0.001
3	*Bacteroides*	P	4.881	0.002
4	*Neisseria*	H	4.707	0.008
5	*Alysiella*	H	4.698	0.010
6	*Corynebacterium*	H	4.655	0.021
7	*Actinomyces*	H	4.067	0.050
8	*Mannheimia*	P	3.955	0.020
9	*Fretibacterium*	P	3.843	0.002
10	*Desulfomicrobium*	P	3.564	0.002
11	*Odoribacter*	P	3.525	0.021
12	*Parvimonas*	P	3.493	0.001
13	*Haemophilus*	H	3.463	0.009
14	*Catabacter*	P	3.458	0.002
15	*Lachnospiraceae_UCG_009*	P	3.453	0.002
16	*Tannerella*	P	3.442	0.004
17	*Methanosphaera*	P	3.427	0.002
18	*Lachnoclostridium*	P	3.426	0.042
19	*Oribacterium*	P	3.362	0.000
20	*FD2005*	P	3.266	0.013
21	*Lampropedia*	P	3.264	0.031
22	*Candidatus_Methanomethylophilus*	P	3.253	0.000
23	*Bilophila*	P	3.248	0.002
24	*Johnsonella*	P	3.223	0.010
25	*Akkermansia*	H	3.180	0.023
26	*Ruminobacter*	P	3.178	0.002
27	*Agathobacter*	P	3.169	0.003
28	*Solobacterium*	P	3.131	0.021
29	*Herbaspirillum*	H	3.127	0.032
30	*Bacillus*	H	3.124	0.032
31	*Christensenellaceae_R_7_group*	P	3.109	0.008
32	*Erysipelatoclostridium*	P	3.093	0.012
33	*Prevotellaceae_UCG_003*	P	3.066	0.009
34	*Alistipes*	P	3.033	0.009
35	*Selenomonas_1*	P	2.999	0.002
36	*Ralstonia*	H	2.943	0.004
37	*Cutibacterium*	H	2.937	0.012
38	*Fastidiosipila*	P	2.901	0.036

39	*Peptostreptococcus*	P	2.891	0.001
40	*Candidatus_Saccharimonas*	P	2.880	0.004
41	*UBA1819*	P	2.876	0.012
42	*Ruminococcaceae_UCG_002*	P	2.849	0.010
43	*Methanobrevibacter*	P	2.814	0.002
44	*Corticibacter*	P	2.813	0.000
45	*Basfia*	P	2.812	0.012
46	*Erysipelothrix*	P	2.793	0.000
47	*Arenimonas*	P	2.792	0.004
48	*Shuttleworthia*	P	2.786	0.012
49	*Flexilinea*	P	2.778	0.000
50	*Diaphorobacter*	H	2.773	0.035
51	*Desulfobulbus*	P	2.773	0.012
52	*Filifactor*	P	2.725	0.002
53	*Hungatella*	P	2.710	0.012
54	*Clostridium_sensu_stricto_1*	H	2.694	0.012
55	*Trichococcus*	H	2.692	0.049
56	*Flavobacterium*	H	2.627	0.001
57	*Anaerobacillus*	H	2.608	0.004
58	*Atopobium*	H	2.589	0.000
59	*Desulfovibrio*	H	2.580	0.027
60	*Staphylococcus*	H	2.573	0.039
61	*Lachnospiraceae_XPB1014_group*	P	2.498	0.002
62	*Peptococcus*	P	2.472	0.009
63	*Fodinicola*	H	2.405	0.012
64	*Rhodoferax*	H	2.366	0.021
65	*Helcococcus*	P	2.365	0.000
66	*Family_XIII_AD3011_group*	P	2.287	0.001

Supplementary 6. Abundance of 18 genera were significantly (LEfSe test with alpha value=0.05 and threshold of absolute logarithmic LDA score>2) altered in both gingivitis (G) versus healthy (H) and Periodontitis-osteomyelitis (P) versus healthy (H) comparisons.

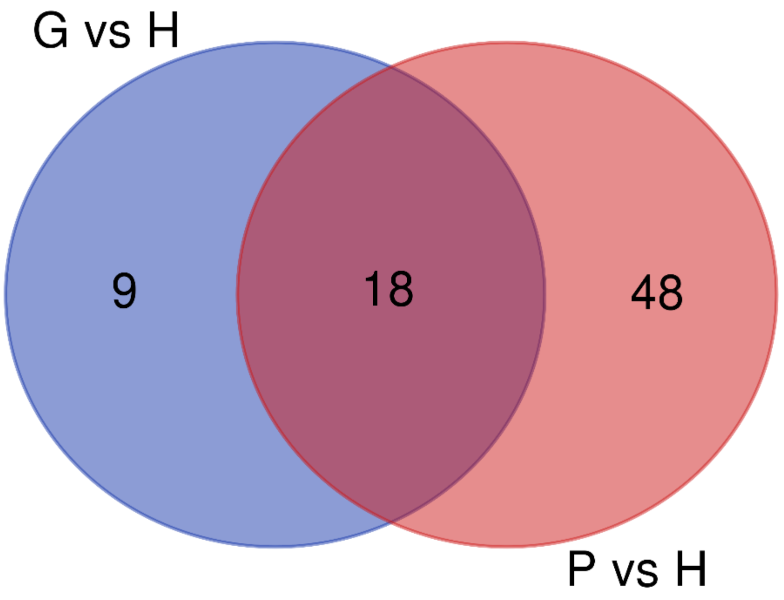

**Comparison**	**Number**	**Genus**
Shared in both gingivitis vs healthy and Periodontitis-osteomyelitis vs healthy comparisons	18	*Porphyromonas*<br>*Fusobacterium*<br>*Bacteroides*<br>*Neisseria*<br>*Desulfomicrobium*<br>*Filifactor*<br>*Corticibacter*<br>*Akkermansia*<br>*Lachnospiraceae_UCG_009*<br>*Ralstonia*<br>*Rhodoferax*<br>*Christensenellaceae_R_7_group*<br>*Johnsonella*<br>*Clostridium_sensu_stricto_1*<br>*Atopobium*<br>*Haemophilus*<br>*Tannerella*<br>*Fretibacterium*
Gingivitis vs healthy specific	9	*Bifidobacterium*<br>*Brevundimonas*

		Suttonella<br>*Dermabacter*<br>*Succiniclasticum*<br>*Musa_itinerans*<br>*Mogibacterium*<br>*Proteocatella*<br>*WCHB1_32*
Periodontitis-osteomyelitis vs healthy specific	48	*Selenomonas_1*<br>*Candidatus_Methanomethylophilus*<br>*Flavobacterium*<br>*Oribacterium*<br>*Peptococcus*<br>*Actinomyces*<br>*Methanosphaera*<br>*Anaerobacillus*<br>*Fastidiosipila*<br>*Lachnoclostridium*<br>*Methanobrevibacter*<br>*FD2005*<br>*Lachnospiraceae_XPB1014_group*<br>*Corynebacterium*<br>*Staphylococcus*<br>*Shuttleworthia*<br>*UBA1819*<br>*Parvimonas*<br>*Bilophila*<br>*Catabacter*<br>*Herbaspirillum*<br>*Alysiella*<br>*Ruminobacter*<br>*Agathobacter*<br>*Lampropedia*<br>*Mannheimia*<br>*Odoribacter*<br>*Peptostreptococcus*<br>*Ruminococcaceae_UCG_002*<br>*Candidatus_Saccharimonas*<br>*Fodinicola*<br>*Hungatella*<br>*Diaphorobacter*<br>*Helcococcus*<br>*Prevotellaceae_UCG_003*

	Cutibacterium
	Alistipes
	Solobacterium
	Flexilinea
	Erysipelatoclostridium
	Desulfobulbus
	Basfia
	Trichococcus
	Arenimonas
	Family_XIII_AD3011_group
	Bacillus
	Erysipelothrix
	Desulfovibrio

Supplementary 7. Formula of Principal Coordinate Analysis (PCoA) of microbiome data of periodontitis-osteomyelitis, gingivitis, and healthy samples. The first component separated periodontitis-osteomyelitis samples as healthy and gingivitis samples, as periodontitis-osteomyelitis samples had the lowest negative coefficient values for this component -0.28, -0.53, -0.39, -0.56, and -0.53.

Sample	PCo 1	PCo 2	PCo 3	PCo 4	PCo 5	PCo 6	PCo 7	PCo 8	PCo 9	PCo 10	PCo 11	….	PCo 27
**G1**	0.09	-0.29	-0.08	0.28	0.02	-0.22	-0.15	-0.06	0.05	0.03	0.00	….	0.00
**G2**	0.10	-0.28	-0.01	0.42	-0.06	-0.05	-0.01	-0.04	0.08	-0.18	0.06	….	0.00
**G3**	0.02	-0.19	0.08	0.00	0.28	0.06	0.10	0.03	0.06	0.03	-0.08	….	0.00
**G4**	-0.45	-0.02	0.06	-0.02	0.20	-0.06	0.18	0.04	0.01	-0.09	0.02	….	0.00
**G5**	0.02	-0.04	0.20	-0.02	0.20	0.00	-0.12	-0.09	-0.22	-0.08	-0.02	….	0.00
**G6**	0.23	0.08	-0.18	-0.14	0.03	-0.06	-0.17	-0.05	0.02	0.02	0.06	….	0.00
**G7**	-0.49	0.05	-0.03	-0.05	-0.11	0.07	-0.05	-0.07	0.04	-0.04	0.04	….	0.00
**H1**	-0.18	-0.08	0.15	0.04	0.30	0.11	-0.07	-0.07	-0.05	0.03	-0.15	….	0.00
**H10**	0.20	0.25	0.23	-0.02	0.03	-0.15	-0.08	0.00	0.02	-0.01	0.08	….	0.00
**H11**	0.14	-0.03	0.41	-0.07	-0.20	-0.05	0.14	-0.10	0.11	0.03	-0.04	….	0.00
**H12**	0.23	-0.05	-0.18	-0.14	0.01	0.14	0.12	-0.22	0.00	-0.07	0.08	….	0.00
**H13**	0.28	0.28	-0.15	0.01	0.06	0.12	0.05	-0.11	0.04	0.01	0.06	….	0.00
**H14**	0.11	0.56	-0.21	0.26	-0.01	-0.15	0.17	0.00	-0.03	0.03	-0.10	….	0.00
**H15**	0.24	0.26	0.07	-0.13	-0.06	-0.08	-0.05	0.04	-0.12	-0.15	0.06	….	0.00
**H2**	0.26	0.32	-0.04	0.11	0.10	0.12	-0.07	0.12	0.04	0.10	-0.05	….	0.00
**H3**	0.19	-0.30	-0.22	0.02	-0.02	0.07	0.02	-0.09	0.01	0.16	0.01	….	0.00
**H4**	0.22	0.04	0.34	-0.08	-0.14	-0.01	0.01	-0.10	0.08	0.11	-0.03	….	0.00
**H5**	0.29	0.08	0.03	-0.04	0.01	0.12	-0.08	0.16	0.06	-0.01	0.02	….	0.00
**H6**	0.24	-0.15	0.08	0.01	-0.08	0.19	-0.04	0.24	0.08	-0.08	0.00	….	0.00
**H7**	0.17	-0.21	-0.19	-0.21	-0.11	-0.23	-0.10	0.05	-0.03	0.04	-0.13	….	0.00
**H8**	0.17	-0.25	0.08	0.12	-0.13	0.01	0.20	0.13	-0.25	0.12	0.10	….	0.00
**H9**	0.22	-0.18	-0.22	-0.20	-0.08	0.08	0.09	0.02	-0.05	-0.13	-0.13	….	0.00
**P1**	-0.28	-0.05	-0.09	-0.10	0.15	0.03	0.00	0.07	0.08	0.08	0.19	….	0.00
**P2**	-0.53	0.06	-0.08	-0.05	-0.10	-0.04	0.06	0.05	0.11	-0.05	-0.08	….	0.00
**P3**	-0.39	-0.03	-0.03	-0.16	0.08	-0.27	0.05	0.08	0.01	0.04	0.06	….	0.00
**P4**	-0.56	0.10	-0.03	0.09	-0.24	0.13	-0.08	-0.03	-0.09	0.06	-0.03	….	0.00
**P5**	-0.53	0.07	0.01	0.06	-0.13	0.13	-0.12	-0.01	-0.06	0.01	0.00	….	0.00

Supplementary 8. Signature of *Porphyromonas*, *Fusobacterium*, and *Bacteroides* in periodontitis-osteomyelitis was robust and consistent in different sexes, species, and zoos.

LDA Effect Size (LEfSe) was used to test relationship between the variables (species, sex, and zoo/location) with and microbiome. The results are added to Supplementary 8. To perform LEfSe test, the class vector was assigned to disease (periodontitis-osteomyelitis verses healthy) and subclass was assigned to variable (such as zoo, species, and sex). LEfSe test was run with alpha value = 0.05 and threshold of absolute logarithmic linear discriminant analysis (LDA) score>2.

Porphyromonas had significantly higher abundance in periodontitis-osteomyelitis (P) samples, irrespective from sex (F: female, M: male). LEfSe test with alpha value = 0.05 and threshold of absolute logarithmic linear discriminant analysis (LDA) score>2.

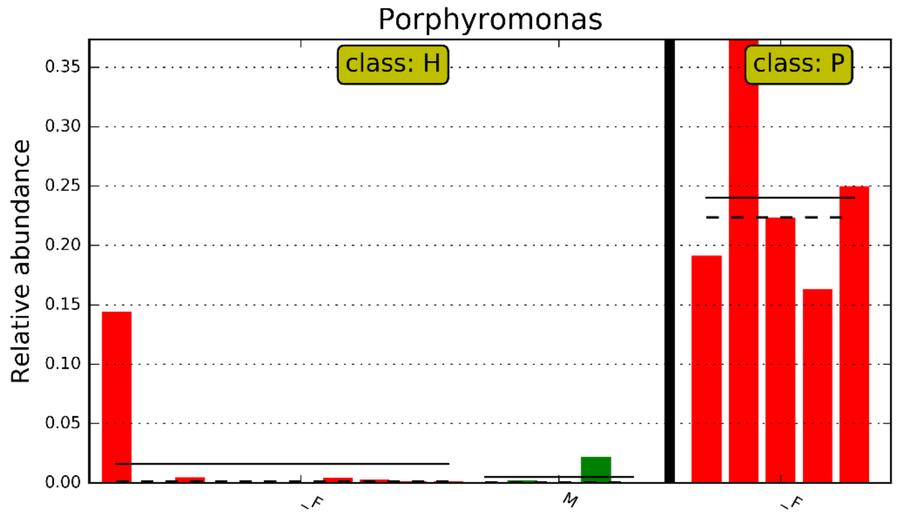

Fusobacterium had significantly higher abundance in periodontitis-osteomyelitis (P) samples, irrespective from sex (F: female, M: male). LEfSe test with alpha value = 0.05 and threshold of absolute logarithmic linear discriminant analysis (LDA) score>2.

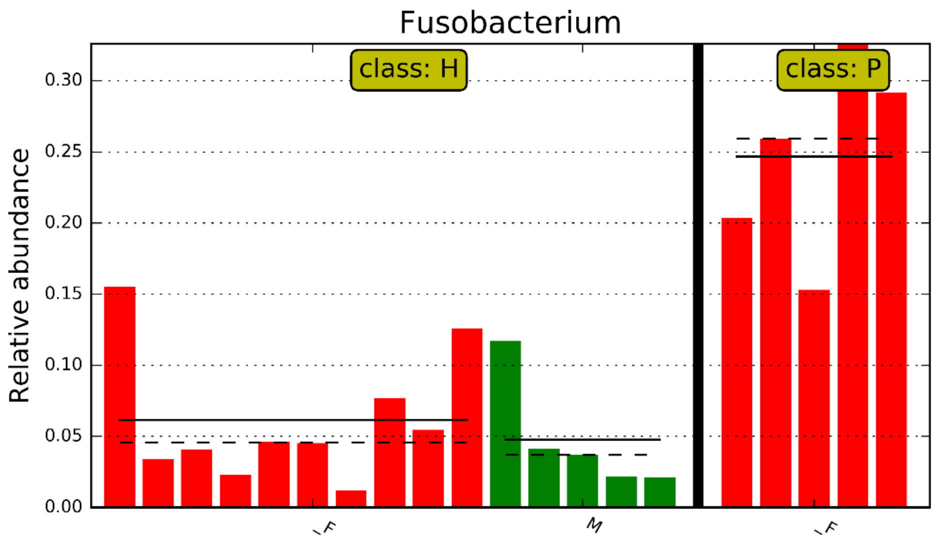

Bacteroides had significantly higher abundance in periodontitis-osteomyelitis (P) samples, irrespective from sex (F: female, M: male). LEfSe test with alpha value = 0.05 and threshold of absolute logarithmic linear discriminant analysis (LDA) score>2.

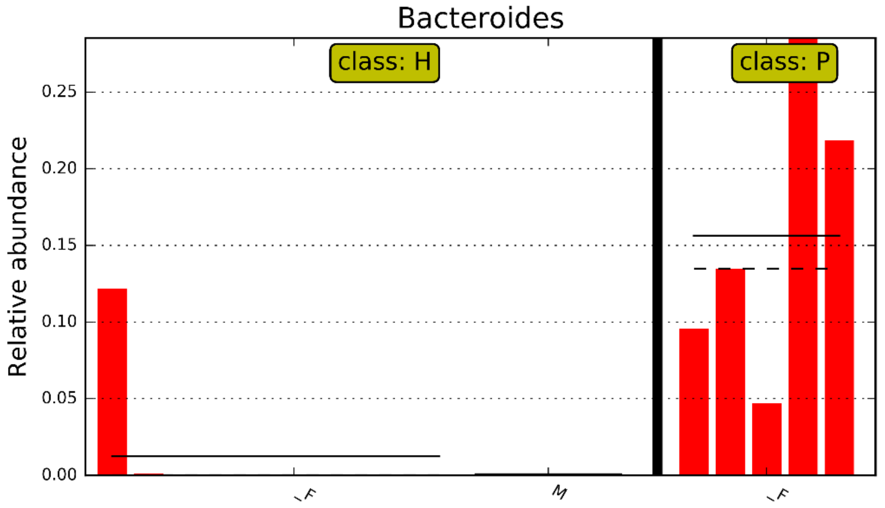

Porphyromonas had significantly higher abundance in periodontitis-osteomyelitis (P) samples, irrespective from zoo location (AZ: Adelaide zoo, MZ: Monarto Zoo). LEfSe test with alpha value = 0.05 and threshold of absolute logarithmic linear discriminant analysis (LDA) score>2.

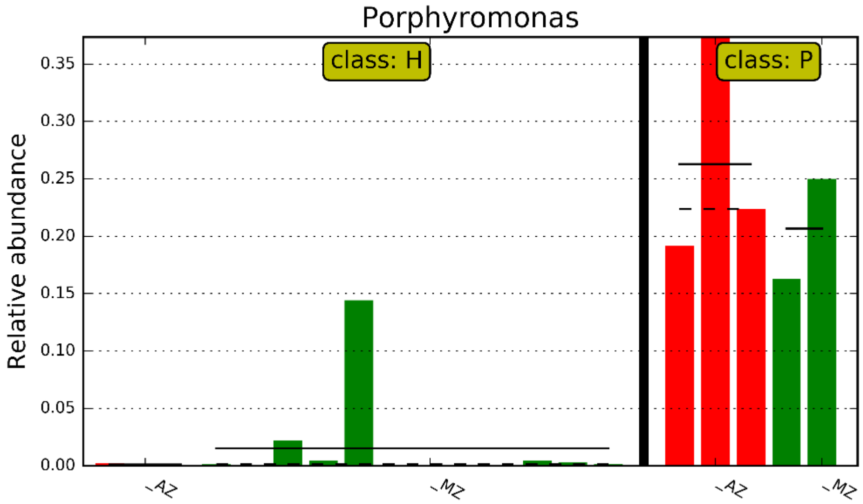

Fusobacterium had significantly higher abundance in periodontitis-osteomyelitis (P) samples, irrespective from zoo location (AZ: Adelaide zoo, MZ: Monarto Zoo). LEfSe test with alpha value = 0.05 and threshold of absolute logarithmic linear discriminant analysis (LDA) score>2.

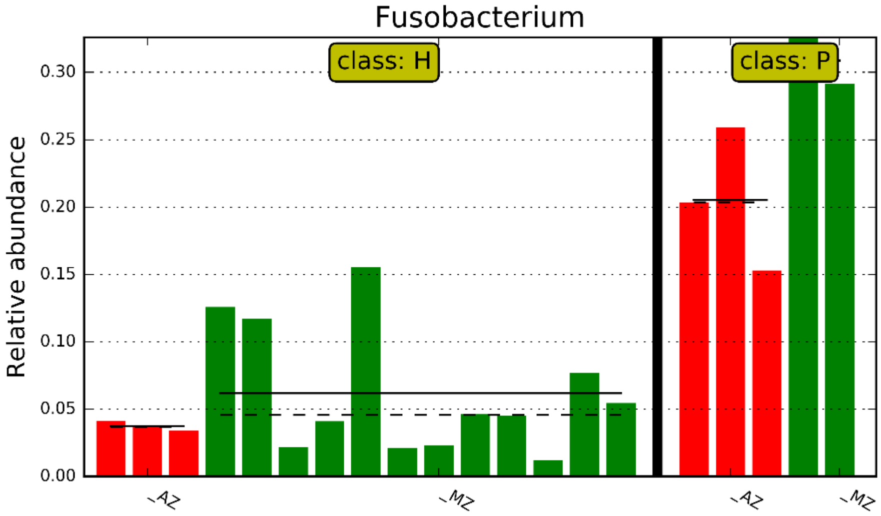

Bacteroides had significantly higher abundance in periodontitis-osteomyelitis (P) samples, irrespective from zoo location (AZ: Adelaide zoo, MZ: Monarto Zoo). LEfSe test with alpha value = 0.05 and threshold of absolute logarithmic linear discriminant analysis (LDA) score>2.

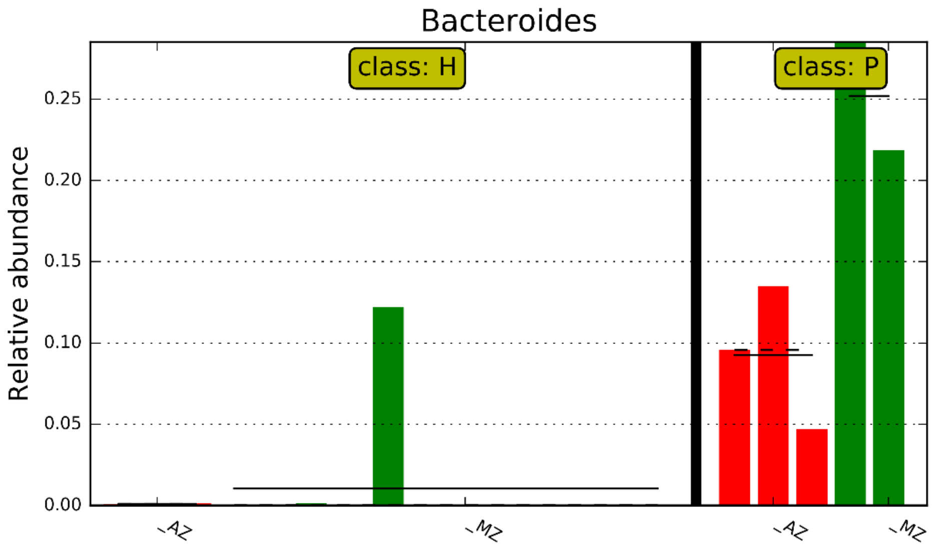

Porphyromonas had significantly higher abundance in periodontitis-osteomyelitis (P) samples, irrespective from species (YFRW: *Petrogale xanthopus*, LNP: *Potorous tridactylus*, TW: *Notamacropus eugenii*). LEfSe test with alpha value = 0.05 and threshold of absolute logarithmic linear discriminant analysis (LDA) score>2.

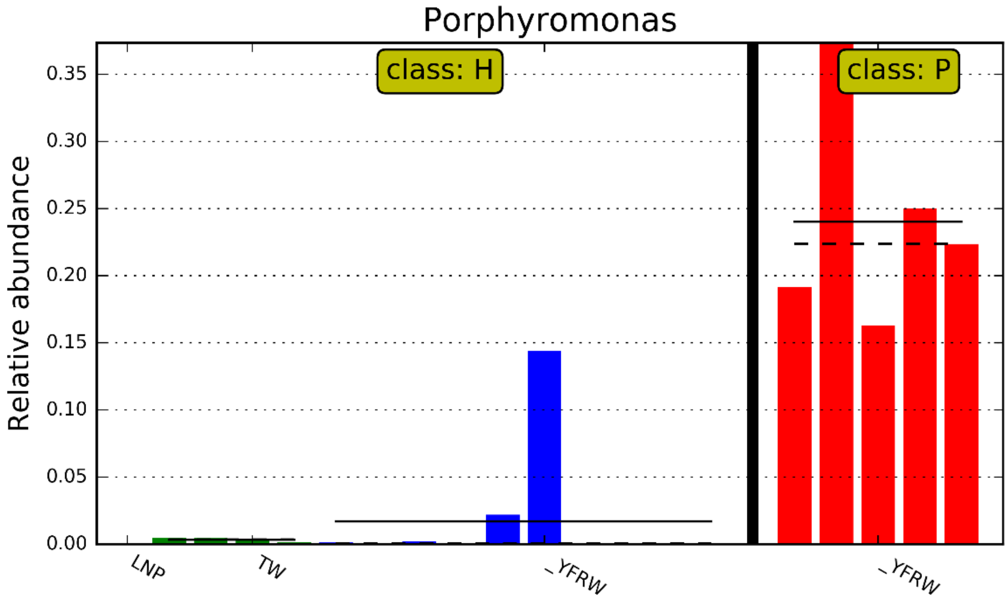

Fusobacterium had significantly higher abundance in periodontitis-osteomyelitis (P) samples, irrespective from species (YFRW: *Petrogale xanthopus*, LNP: *Potorous tridactylus*, TW: *Notamacropus eugenii*). LEfSe test with alpha value = 0.05 and threshold of absolute logarithmic linear discriminant analysis (LDA) score>2.

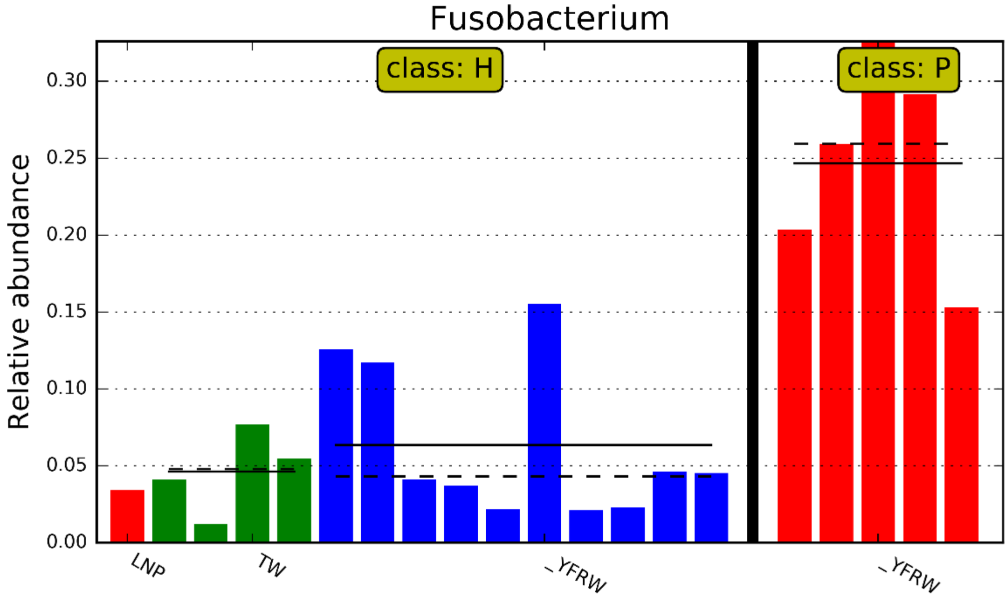

Bacteroides had significantly higher abundance in periodontitis-osteomyelitis (P) samples, irrespective from species (YFRW: *Petrogale xanthopus*, LNP: *Potorous tridactylus*, TW: *Notamacropus eugenii*). LEfSe test with alpha value = 0.05 and threshold of absolute logarithmic linear discriminant analysis (LDA) score>2.

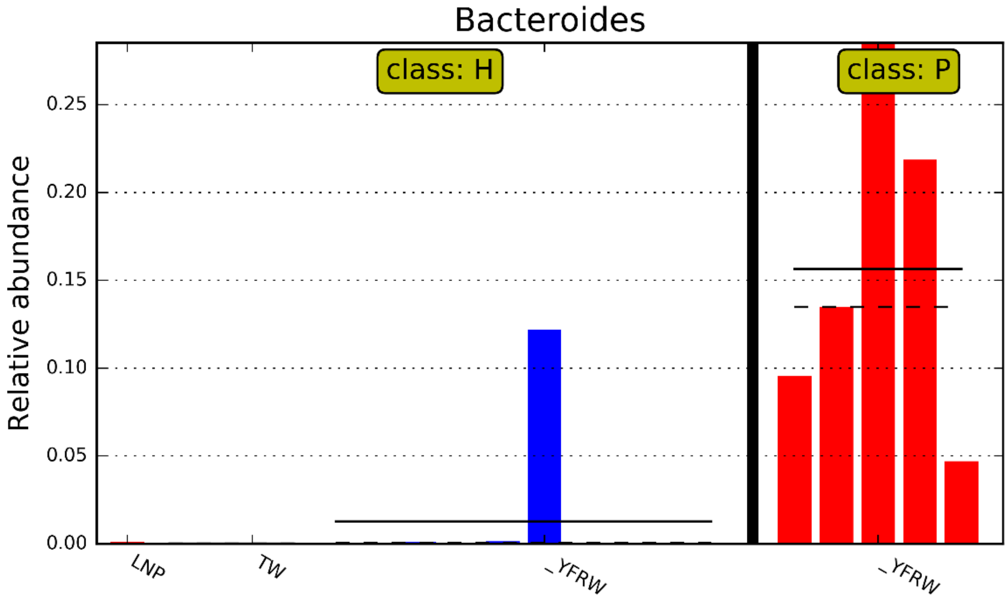

Supplementary 9. Meta-data of experiment.

**Health Status**	**Label**	**Sample ID**	**Animal**	**Number**	**Final label**	**Number**	**Sample ID**	**Sample ID**	**Zoo**	**Species**	**Sex**	**Age**	**Weight**	**Label**
Gingivitis	G1	Y19_S78	16	1	G1	**NO. G = 7**	Y19	Y19	MZ	TW	F	2	3.36	G1
Gingivitis	G2	Y31_S113	16	2	G2		Y31	Y31	MZ	TW	F	2	3.36	G2
Gingivitis	G3	Y20_S79	17	3	G3		Y20	Y20	AZ	YFRW	F	2.25	5.84	G3
Gingivitis	G4	Y32_S89	17	4	G4		Y32	Y32	AZ	YFRW	F	2.25	5.84	G4
Gingivitis	G5	Y21_S80	18	5	G5		Y21	Y21	AZ	YFRW	F	9.25	7.24	G5
Gingivitis	G6	Y33_S114	18	6	G6		Y33	Y33	AZ	YFRW	F	9.25	7.24	G6
Gingivitis	G7	Y27_S86	19	7	G7		Y27	Y27	MZ	Dead	Dead	Dead	Dead	G7
Healthy	H1	Y1_S67	1	1	H1	**NO. H = 15**	Y1	Y1	MZ	YFRW	F	12.5	6.59	H1
Healthy	H2	Y2_S68	2	2	H2		Y2	Y2	MZ	YFRW	M	1.5	5.57	H2
Healthy	H4	Y4_S116	3	3	H3		Y4	Y4	MZ	YFRW	F	0.75	3.275	H4
Healthy	H5	Y5_S117	4	4	H4		Y5	Y5	MZ	YFRW	F	2.25	5.97	H5
Healthy	H6	Y6_S118	5	5	H5		Y6	Y6	MZ	YFRW	F	2	5.47	H6
Healthy	H7	Y7_S69	6	6	H6		Y7	Y7	MZ	TW	F	2	4.75	H7
Healthy	H8	Y8_S119	7	7	H7		Y8	Y8	MZ	TW	F	1.25	2.85	H8
Healthy	H9	Y9_S70	8	8	H8		Y9	Y9	MZ	TW	F	1.25	2.79	H9
Healthy	H10	Y10_S71	9	9	H9		Y10	Y10	MZ	YFRW	F	11.75	7.185	H10
Healthy	H11	Y11_S72	10	10	H10		Y11	Y11	MZ	YFRW	M	1.5	4.77	H11

Healthy	H12	Y12_S 73	11	11	H11		Y12	Y12	AZ	YFR W	M	1.25	5.16	H12
Healthy	H13	Y13_S 74	12	12	H12		Y13	Y13	AZ	YFR W	M	1	4.44	H13
Healthy	H14	Y14_S 75	13	13	H13		Y14	Y14	AZ	LNP	F	11	1.086	H14
Healthy	H15	Y15_S 76	14	14	H14		Y15	Y15	MZ	YFR W	M	3.5	8.53	H15
Healthy	H18	Y18_S 77	15	15	H15		Y18	Y18	MZ	TW	F	3	3.81	H18
Periodontitis-osteomyelitis	P1	Y22_S 81	20	1	P1	**NO. P = 5**	Y22	Y22	AZ	YFR W	F	9	6.54	P1
Periodontitis-osteomyelitis	P2	Y24_S 83	21	2	P2		Y24	Y24	AZ	YFR W	F	4.25	6.54	P2
Periodontitis-osteomyelitis	P3	Y34_S 90	21	3	P3		Y34	Y34	AZ	YFR W	F	4.25	6.54	P3
Periodontitis-osteomyelitis	P4	Y28_S 87	22	4	P4		Y28	Y28	MZ	YFR W	F	9.75	6.1	P4
Periodontitis-osteomyelitis	P5	Y29_S 88	22	5	P5		Y29	Y29	MZ	YFR W	F	9.75	6.1	P5
**Total**	**27 samp les**		**22 Anim als**											
